# Gender differences in knowledge, attitudes, and practices with respect to type 1 diabetes among Saudi public-school teachers

**DOI:** 10.1186/s12889-023-15043-w

**Published:** 2023-01-17

**Authors:** Najlaa M. Aljefree, Noha M. Almoraie, Maha A. Althaiban, Mahitab A. Hanbazaza, Huda A. Wazzan, Israa M. Shatwan

**Affiliations:** Food and Nutrition Department, Faculty of Human Sciences and Design, King Abdulaziz University, Jeddah, 3270 Saudi Arabia

**Keywords:** Type 1 diabetes, Children, Knowledge, Attitudes, Practice, School teachers, Gender, Jeddah city

## Abstract

**Background:**

Children with type 1 diabetes mellitus (T1DM) need carefully monitoring even during school hours to avoid emergencies. Hence, it is crucial for teachers to have appropriate knowledge and positive attitudes toward diabetes to effectively manage the disease and its complications. This study aimed to compare the knowledge, attitudes, and practices with respect to T1DM among Saudi male and female public-school teachers living in Jeddah and to identify the associated factors.

**Methods:**

A cross-sectional study was conducted among primary, intermediate, and secondary public-school teachers working in Jeddah, Saudi Arabia between October 2021 and March 2022. Data were collected through an online survey. The survey included information on the socio-demographics of school teachers, the presence of chronic diseases, teachers’ health behaviours, and knowledge, attitude, and practice with respect to T1DM.

**Results:**

This study included 378 school teachers. The majority of school teachers from both genders were married, held a bachelor’s degree, and aged 45–54 years. Male school teachers were married (*P* = 0.02), held postgraduate certificates (*P* < 0.001), smoked cigarette and shisha (*P* < 0.001), and were physically active (*P* < 0.001) compared to female school teachers. Regarding teachers’ knowledge of T1DM, it was found that female teachers were significantly more knowledgeable of T1DM children’s and its symptoms than male teachers (*P* = 0.03; *P* = 0.01, respectively). However, male teachers were more willing to accommodate T1DM students in their classes and attend programs to support T1DM students as compared to female teachers (*P* = 0.004; *P* = 0.004, respectively). Moreover, the study showed poor practice scores for T1DM. In addition, the knowledge, attitudes, and practice scores toward T1DM were significantly associated with advanced age (*P* = 0.002), and extended years of teaching experience (*P* ≤ 0.002). Also, diabetic teachers had the highest knowledge (*P* = 0.03) and attitude (*P* = 0.02) scores compared to non-diabetic teachers. Male teachers who were married (*P* = 0.002), shisha smokers (*P* = 0.01), and had never practiced activity (*P* = 0.03) had better attitudes and practices toward T1DM. Similarly, female teachers who held bachelor’s certificates had better attitudes toward T1DM (*P* = 0.02).

**Conclusion:**

The present study revealed moderate knowledge, favorable attitudes, and poor practices related to T1DM among school teachers in Jeddah. It is crucial for policymakers to provide school teachers with training for necessary diabetes care for diabetic students.

**Supplementary Information:**

The online version contains supplementary material available at 10.1186/s12889-023-15043-w.

## Introduction

Diabetes mellitus is a chronic metabolic disease characterised by an increase in the blood sugar level, caused by insufficient insulin production by the pancreas (type 1 diabetes mellitus) or by insulin resistance in the cells (type 2 diabetes mellitus) [1]. According to the World Health Organization (WHO) by 2025, approximately 300 million people will be diagnosed with either type 1 or type 2 diabetes worldwide [2]. In addition, diabetes significantly increases the risk of heart disease, stroke, end-stage renal disease, dialysis, and blindness in the affected population [3]. The incidence of diabetes is increasing globally, especially in developing countries, because of decreased physical activity and increased availability of calorie-dense foods [4]. Type 1 diabetes mellitus (T1DM) is the most prevalent type of diabetes in children and adolescents [5]. It occurs between the ages of 10 and 15 years, and three-quarters of cases are diagnosed before the age of 18 years [6]. In many countries, the prevalence of T1DM in children is rising; current estimates imply that 79,000 children under the age of 15 develop T1DM annually worldwide [7]. T1DM affects 31.4 new children per 100,000 of total population in Saudi Arabia every year. Saudi Arabia has the fifth highest annual rate of T1DM incidence globally according to the recent International Diabetes Federation report [8]. The total number of children in the country with T1DM is 16,100, which is a quarter of the total diabetic children population in the Middle East region (60,700) [9]. According to a study conducted in Jeddah, Saudi Arabia, 157 children (68.9%) experienced hypoglycaemia crises every year [10]. These numbers indicate the increasing incidence of T1DM among children in Saudi Arabia and the increased need for appropriate diabetes management, care, and awareness [5].

Schools are an important part of every child’s life. Childhood diabetes has a significant impact on academic performance [7]. Furthermore, children with diabetes are at a high risk for emotional outbursts and behavioural disorders which could hinder academic achievements [11]. Since T1DM is predominant among school children, appropriate awareness about the disease should be imparted in school teachers. Children with diabetes require experienced teachers to assist them in controlling and managing their diabetes at school and performing their daily activities [12]. Students with diabetes frequently face emergencies, such as hypoglycaemia and hyperglycaemia; thus, the educational environment needs immediate access to provide care for these students [13, 14]. Accordingly, teachers should be provided with sufficient training to manage diabetes related emergencies. Few studies have assessed the knowledge, attitudes, and practices of school teachers with respect to T1DM in Saudi Arabia [5]. Therefore, the current study aimed to examine the knowledge, attitudes, and practice with respect o T1DM among Saudi public school teachers living in Jeddah and studied the gender differences. Moreover, the study aimed to identify factors associated with these differences.

## Methods

### Study design and participants

This cross-sectional study was conducted in Jeddah, Saudi Arabia between October 2021 and March 2022. This study was conducted in accordance with the guidelines of the Declaration of Helsinki and approved by the Biomedical Ethics Research Committee of King Abdul-Aziz University (Reference No. 159–21). The study was approved by the Education Department of Jeddah City. There are six administrative educational offices for public schools: North Jeddah, East, Centre, Naseem, South, and Safa. The sample size was calculated based on the number of male and female teachers at public schools in Jeddah. There were 6046, 3777, and 2189 male teachers in primary, intermediate, and secondary public schools, respectively. Likewise, there were 7304, 4323, and 3219 female teachers in primary, intermediate, and secondary public schools, respectively [15]. Hence, the sample size required to achieve sufficient statistical power was 378 participants. It was determined within 0.05 of the total population of (26,858 teachers) with a 95% confidence level, a 5% margin error, and a response distribution of 50% [16]. The inclusion criteria were school teachers of both genders who were working in primary, intermediate, and secondary public schools in Jeddah. After obtaining ethical approval, the online surveys were sent to various educational offices which then distributed them to public schools in their area. The distribution of teacher included in the study were as follows: North Jeddah (29.4%), East (16.1%), Centre (19.8%), South (22.5%), Asafa (6.3%), and Naseem (5.8%).

### Data collection

Data were collected through an online survey during school work. The questionnaire was composed of six sections: socio-demographic characteristics of school teachers, the presence of chronic diseases, participants’ health behaviours, knowledge, attitude, and practices with respect to T1DM. The survey was adapted from previous studies [5]. For reliability and validity, the researchers translated the survey into Arabic language, and pre-testing was done after that among 15 school teachers to ensure that no questions appeared complex or were misinterpreted. At the beginning of the online survey, school teachers were informed about the research objectives and procedures and guaranteed their anonymity and voluntary participation in this research. Additionally, those who agreed to participate completed the survey.

### Socio-demographic and health data of school teachers

In the first section of the survey, socio-demographic factors such as gender, age, social status, education level, major, school level, educational office to which the school belongs, and years of teaching experience were collected from the teachers. The second section included questions related to the presence of chronic diseases, such as diabetes, coronary heart disease, hypertension, and high cholesterol levels. The third section comprised of questions regarding health behaviours including smoking (cigarettes or shisha) and physical activity. They were categorised into three groups based on their cigarette smoking habits (current smoker, past smoker, and non-smoker) [17]. Similarly, shisha smoking habits were categorised as (regular smokers, rare smokers, never) [18]. In addition, participants were asked to report their physical activity, and were classified into moderately active (never, rarely, 1–2 per week, 3–4 per week, more than 5 per week) and very active (never, rarely, 1–2 per week, 3–4 per week, more than 5 per week) [19].

### Knowledge, attitude, and practice toward T1DM questionnaire

Questions related to knowledge, attitude, and practice with respect to T1DM were adapted from validated questionnaires [5] (Additional file 1). The questions on knowledge, attitudes and practices with three possible responses: “Yes”, “No”, and “Not sure”. Each correct answer was awarded a score of 1 point, the correct answer was “yes”. While the incorrect answers, “No”, and “Not sure”, were awarded a score of 0. Participants were considered to have poor knowledge, unfavorable attitude, and poor practices with respect to T1DM when they have a score of less than 60% of the total scores [20, 21]. Regarding the questionnaire, the fourth section comprised of questions about knowledge that contained 7 structured questions on knowledge about T1DM. The questions specifically covered school teachers’ knowledge about the symptoms of T1DM, complications, and treatment for T1DM. The total calculated knowledge score ranged from to 0–7. The knowledge scores were categorized as poor (0 - < 4), moderate (4 - < 5), good (≥5–7). Attitude was described in section five of the questionnaire and contained 5 structured questions. The total calculated scores for attitudes ranged from to 0–5. Attitude scores were classified as unfavorable (0 - < 3), or favorable (≥3–5). Practices related to T1DM were described in Section 6 of the questionnaire, which contains 10 structured questions. The total cumulative scores for the practices ranged from to 0–10. The practice scores were categorized as poor (0 - < 6), moderate (6 - < 8), good (≥8–10).

### Statistical analysis

The SPSS software (version 28) was used for statistical analysis. The data are presented in tables and figures as frequency and percentage for sociodemographic and health data, and answers for knowledge, attitudes, and practice toward T1DM questions and mean ± SD for scores. The chi-square test was used to determine significant differences in qualitative variables. We performed a linear regression test to assess the association between sociodemographic and health data as well as knowledge, attitudes, and practice toward T1DM scores. Statistical significance was set to 0.05.

## Results

### Socio-demographic characteristics and health data of school teachers

A total of 378 school teachers participated in this study. The sociodemographic characteristics and health data of the school teachers stratified by gender are shown in Table 1. The majority of the teachers were aged 45–54 years (48.8 and 39.5% female and male, respectively), followed by age group 35–44 years (31.8 and 33.3% female and male, respectively), and had more than 20 years of teaching experience (41.8 and 37.9% female and male, respectively). Secondary and primary school teachers were the main contributors to this study. There were significant gender differences in teachers’ marital status and educational qualifications. The majority of teachers (82.2%) from both genders had a bachelor’s degree as their highest educational qualification. The proportion of male teachers holding postgraduate degrees was higher than that of females (12.4% vs. 3.5%), while the proportion of females holding teaching diplomas was higher than that of males (*P* < 0.001). The majority of school teachers from both genders were married, the percentage of single males was higher than that of females, and the percentage of divorced females was higher than that of males (*P* = 0.02). The highest percentage of school teachers’ majors in this study were Arabic language teachers (16.9%), followed by science teachers (14.8%), Islamic religions (14.6%), and math teachers (11.9%) (data not shown). Based on the health data findings, there were significant differences between sexes in smoking status. The number of non-smoking (cigarette) female teachers was higher than that of non-smoking male teachers (*P* < 0.001). The same trend was observed for smoking shisha with the number of female teachers that never smoked higher (*P* < 0.001) than the number of male teachers that never smoked. However, male teachers had a more active lifestyle as compared to female teachers (*P* < 0.001).

**Table 1:** Socio-demographic characteristics and health data of the schoolteachers stratified by gender

Characterization	Male (*n* = 177) n (%)	Female (*n* = 201) n (%)	*P* value
Age			
20–24	7 (4)	12 (6)	0.11
25–34	18 (10.2)	12 (6)	
35–44	59 (33.3)	64 (31.8)	
45–54	70 (39.5)	98 (48.8)	
55–62	23 (13)	15 (7.5)	
Marital status			
Single	24 (13.6)	20 (10)	**0.02**
Married	142 (80.2)	156 (77.6)	
Divorced	11 (6.2)	20 (10)	
Widow	0	5 (2.5)	
Education level			
Diploma	11 (6.2)	27 (13.4)	**< 0.001**
Bachelor	144 (81.4)	167 (83.1)	
Postgraduate	22 (12.4)	7 (3.5)	
School stage			
Primary	65 (36.7)	74 (36.8)	0.97
Intermediate	45 (25.4)	53 (26.4)	
Secondary	67 (37.9)	74 (36.8)	
Experience years			
1–5	29 (16.4)	20 (10)	0.31
6–10	21 (11.9)	32 (15.9)	
11–15	33 (18.6)	33 (16.4)	
16–20	27 (15.3)	32 (15.9)	
Above 20	67 (37.9)	84 (41.8)	
Health condition			
Healthy	109 (61.6)	115 (57.2)	0.41
Diabetes	26 (14.7)	32 (15.9)	
Heart disease	6 (3.4)	3 (1.5)	
Hypertension	21 (11.9)	24 (11.9)	
High cholesterol	15 (8.5)	27 (13.4)	
Smoking cigarette			
Smoker	47 (26.6)	5 (2.5)	**< 0.001**
Past smoker	29 (16.4)	0	
Non-smoker	101 (57.1)	196 (97.5)	
Smoking Shisha			
Regular smoker	15 (8.5)	4 (2)	**< 0.001**
Never	123 (69.5)	178 (88.6)	
Rarely smoker	39 (22)	19 (9.5)	
Moderate physical activity			
Never	18 (10.2)	22 (10.9)	0.08
Rarely	52 (29.4)	83 (41.3)	
1–2 per week	55 (31.1)	57 (28.4)	
3–4 per week	42 (23.7)	33 (16.4)	
More than 5 per week	10 (5.6)	6 (3)	
Sever activity			
Never	50 (28.2)	92 (45.8)	**< 0.001**
Rarely	66 (37.3)	73 (36.3)	
1–2 per week	33 (18.6)	26 (12.9)	
3–4 per week	23 (13)	9 (4.5)	
More than 5 per week	5 (2.8)	1 (0.5)	

### Knowledge related to T1DM

**Fig. 1:**
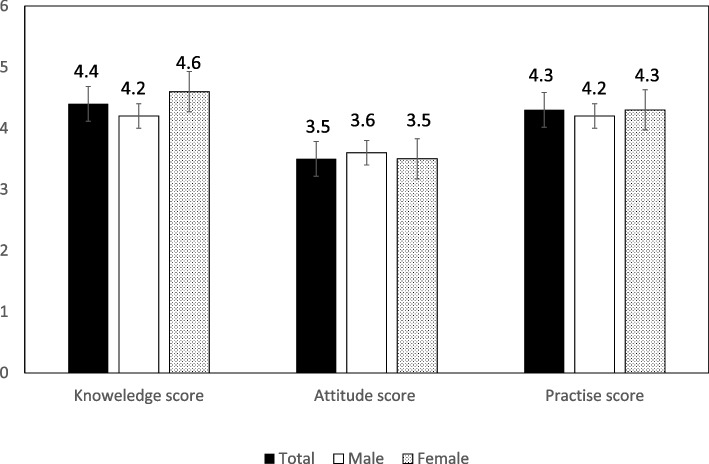
The average knowledge, attitude, and practice scores for T1DM stratified by gender

School teachers’ responses to knowledge toward T1DM stratified by gender are presented in Table 2. The number of female teachers who correctly answered the question regarding onset age of type 1 disease was significantly higher than that of male teachers (*P* = 0.03). Additionally, the percentage of female teachers reporting symptoms of T1DM included fatigue, and the loss of concentration was higher than that of male teachers (*P* = 0.01). There were no other significant gender differences with respect to the questions on knowledge. The average knowledge score (Fig. 1) acquired by all teachers was 4.4 (male teachers: 4.2; female teachers: 4.6).

**Table 2:** School teachers’ knowledge of T1DM stratified by gender

Knowledge Question	Male			Female			*P* value
	Yes n (%)	No n (%)	Not sure n (%)	Yes n (%)	No n (%)	Not sure n (%)	
Students can have T1DM	67 (37.9)	23 (13)	87 (49.2)	85 (42.4)	11 (5.5)	105 (52.2)	**0.03**
T1DM leads to polyuria in diabetic student	147 (83.1)	2 (1.1)	28 (15.8)	178 (88.6)	2 (1)	21 (10.4)	0.29
T1DM leads to fatigue and lack of concentration in diabetic student	136 (76.8)	8 (4.5)	33 (18.6)	177 (88.1)	5 (2.5)	19 (9.5)	**0.01**
T1DM leads to loss of weight in diabetic student	100 (56.5)	22 (12.4)	55 (31.1)	131 (65.2)	17 (8.5)	53 (26.4)	0.18
T1DM is treated with insulin	92 (52)	18 (10.2)	67 (37.9)	109 (54.2)	31 (15.4)	61 (30.3)	0.16
Tremors and sweating means hypoglycaemia in diabetic student	130 (73.4)	5 (2.8)	42 (23.7)	155 (77.1)	2 (1)	44 (21.9)	0.36
The diabetic student should take sweets or juices before physical activities class	82 (46.3)	30 (16.9)	65 (36.7)	90 (44.8)	36 (17.9)	75 (37.3)	0.94

T1DM: type 1 diabetes mellitus

### Attitude toward T1DM

School teachers’ responses to attitudes toward T1DM stratified by gender are presented in Table 3. The male teachers (61%) were more willing to have T1DM students in their classes than were female teachers (52.2%; *P* = 0.04). Regarding the willingness to attend training program to support T1DM students, 73% of male teachers answered yes compared to 57.7% of female teachers (*P* = 0.004). In case of coma or hypoglycemia, the majority of teachers from both sexes stated that they would give the students products with sugar-added in them. In addition, almost all teachers supported the presence of school nurses. The average attitude score (Fig. 1) acquired by all teachers was 3.5 (male teachers: 3.6; female teachers: 3.5).

**Table 3:** School teachers’ attitudes toward T1DM stratified by gender

Attitude Question	Male			Female			*P* value
	Yes n (%)	No n (%)	Not sure n (%)	Yes n (%)	No n (%)	Not sure n (%)	
Are you willing to have diabetic children in your class	108 (61)	35 (19.8)	34 (19.2)	105 (52.2)	62 (30.8)	34 (16.9)	**0.04**
In case of hypoglycaemia, should the diabetic student take sweet juice?	151 (85.3)	6 (3.4)	20 (11.3)	176 (87.6)	4 (2)	21 (10.4)	0.66
In case of coma, can small amount of jam or honey be put into the mouth of the diabetic student?	93 (52.5)	18 (10.2)	66 (37.7)	124 (61.7)	24 (11.9)	53 (26.4)	0.07
Would you like to join training program for dealing with diabetic students?	129 (72.9)	25 (14.1)	23 (13)	116 (57.7)	54 (26.9)	31 (15.4)	**0.004**
Do you support presence of school nurse?	161 (91)	5 (2.8)	11 (6.2)	187 (93)	9 (4.5)	5 (2.5)	0.14

### Practices related to T1DM

School teachers’ responses to practices related to T1DM questions stratified by gender are presented in Table 4. Of the female teachers, 70% of them stated that T1DM was associated with an increased rate of absence among students as compared to the 54.8% of male teachers (*P* < 0.001). A total of 161 (80.1%) female teachers declared that they would give support to T1DM students, compared to 123 (69.5%) male teachers (*P* = 0.04). The average practice score (Fig. 1) acquired by all teachers was 4.3 (male teachers: 4.2; female teachers: 4.3).

**Table 4:** School teachers’ responses to practices related to T1DM questions stratified by gender

Practices Question	Male			Female			*P* value
	Yes n (%)	No n (%)	Not sure n (%)	Yes n (%)	No n (%)	Not sure n (%)	
T1DM affects the student’ academic performance	101 (57.1)	37 (20.9)	39 (22)	126 (62.7)	45 (22.4)	30 (14.9)	0.20
T1DM increases absence rate of diabetic student	97 (54.8)	37 (20.9)	43 (24.3)	141 (70.1)	39 (19.4)	21 (10.4)	**< 0.001**
Do you have any diabetic student in your class?	66 (37.3)	77 (43.5)	34 (19.2)	65 (32.3)	105 (52.2)	31 (15.4)	0.23
Do you give support to the diabetic children in your class?	123 (69.5)	20 (11.3)	34 (19.2)	161 (80.1)	12 (6)	28 (13.9)	**0.04**
Are diabetic children eligible to attend the physical education session?	112 (63.3)	8 (4.5)	57 (32.2)	131 (65.2)	18 (9)	52 (25.9)	0.13
Does your school present special meals for diabetic student?	18 (10.2)	122 (68.9)	37 (20.9)	13 (6.5)	153 (76.1)	35 (17.4)	0.24
Does your school appoint somebody to look after the diabetic students?	78 (44.1)	64 (36.2)	35 (19.8)	101 (50.2)	67 (33.3)	33 (16.4)	0.45
Is there any trained person to check blood sugar and inject insulin in your school?	54 (30.5)	80 (45.2)	43 (24.3)	50 (24.9)	106 (52.7)	45 (22.4)	0.31
Is there any trained person in dealing with diabetic emergencies in your school?	61 (34.5)	69 (39)	47 (26.6)	57 (28.4)	90 (44.8)	54 (26.9)	0.39
Does your school have a training program for dealing with diabetic students?	40 (22.6)	96 (54.2)	41 (23.2)	37 (18.4)	113 (56.2)	51 (25.4)	0.58

T1DM: type 1 diabetes mellitus

### Factors associated with knowledge, attitude, and practices scores toward T1DM

The association between knowledge, attitude, and practice scores for T1DM and school teachers’ socio-demographics and health data stratified by gender is presented in Table 5. There was a significant positive association between the increase in age of male school teachers and attitudes (*P* = 0.02) and practices (*P* < 0.001) scores. Likewise, there was a positive association between an increase in age and knowledge score among male teachers; however, the difference was barely significant (*P* = 0.05). Male teachers aged 55 to 62 years had scores for attitude and practice of 3.9 ± 1.1 and 5.1 ± 2.4, respectively compared to male teachers aged 20 to 24 years with scores of 2.4 ± 2.1 and 1.4 ± 1.9, respectively. Among female teachers, only the attitude score showed a positive association with age (*P* = 0.002). Married male teachers had higher attitude scores than single and divorced teachers (*P* < 0.001), while divorced male teachers had higher practice scores than married and single teachers (*P* < 0.001). In addition, married male teachers had higher knowledge scores than single and divorced teachers; however, the difference was barely significant (*P* = 0.05). However, marital status did not show any significant association with knowledge, attitude, or practice scores among female teachers.

**Table 5:** Association between knowledge, attitude, and practice scores toward T1DM and school teachers’ socio-demographics and health data stratified by gender

Characteristics	Male			Female		
	Knowledge	Attitude	Practices	Knowledge	Attitude	Practices
Age						
20–24	2.5 ± 1.7	2.4 ± 2.1	1.4 ± 1.9	4 ± 1.8	3.2 ± 1.4	3.3 ± 1.6
25–34	3.7 ± 1.8	3.2 ± 1.1	2.1 ± 2.1	3.5 ± 2.6	2.3 ± 1.3	3.3 ± 1.8
35–44	4.2 ± 1.8	3.5 ± 1.2	4.5 ± 2.3	4.8 ± 1.7	3.5 ± 1.1	4.3 ± 2
54–54	4.4 ± 1.7	3.8 ± 1.2	4.5 ± 2.0	4.6 ± 1.5	3.6 ± 0.9	4.6 ± 2.1
55–62	4.6 ± 1.7	3.9 ± 1.1	5.1 ± 2.4	4.5 ± 1.8	3.7 ± 0.7	4.4 ± 2.4
*P* value	**0.05**	**0.02**	**< 0.001**	0.10	**0.002**	0.09
Marital status						
Single	3.4 ± 1.9	3 ± 1.4	2.1 ± 2.3	4.3 ± 1.7	3.3 ± 1.3	3.6 ± 1.4
Married	4.4 ± 1.8	3.8 ± 1.1	4.5 ± 2.1	4.6 ± 1.7	3.5 ± 1.1	4.5 ± 2.1
Divorced	3.9 ± 1.9	2.6 ± 1.6	4.6 ± 3.2	4.2 ± 1.5	3.3 ± 1	4.1 ± 2
Widow	0	0	0	4.6 ± 1.5	3.4 ± 0.5	4.4 ± 1.3
*P* value	**0.05**	**< 0.001**	**< 0.001**	0.60	0.73	0.28
Education level						
Diploma	4 ± 2.1	3.4 ± 1.8	3.2 ± 2.2	4.6 ± 2.1	3.1 ± 1.2	4.2 ± 2.2
Bachelor	4.3 ± 1.8	3.7 ± 1.1	4.4 ± 2.3	4.6 ± 1.6	3.6 ± 1.1	4.4 ± 2.1
Postgraduate	3.8 ± 1.9	3.1 ± 1.4	3.6 ± 2.1	3.7 ± 1.7	3 ± 1.4	4.1 ± 1.8
*P* value	0.48	0.17	0.13	0.38	**0.02**	0.88
School stage						
Primary	4.6 ± 1.6	3.6 ± 1.3	4.4 ± 2.2	4.7 ± 1.7	3.5 ± 1.1	4.2 ± 2
Secondary	3.8 ± 1.7	3.5 ± 1.2	4.1 ± 2.4	4.6 ± 1.5	3.6 ± 1	4.7 ± 2.1
High	4.1 ± 2.1	3.6 ± 1.1	4.1 ± 2.4	4.3 ± 1.8	3.3 ± 1.2	4.3 ± 2.1
*P* value	0.09	0.77	0.61	0.36	0.45	0.35
Experience years						
1–5	3.7 ± 2	3.1 ± 1.3	2.8 ± 2.8	3.9 ± 1.9	3.2 ± 1.3	2.9 ± 1.4
6–10	4.1 ± 2.1	3.6 ± 1.1	3.9 ± 2.6	4.4 ± 1.7	3.4 ± 1.3	4.4 ± 2.1
11–15	4.3 ± 1.3	3.7 ± 1	4.8 ± 2.2	4.6 ± 1.7	3.3 ± 1.1	4.3 ± 1.9
16–20	3.2 ± 2	2.9 ± 1.5	3.7 ± 1.9	4.7 ± 1.8	3.4 ± 0.9	4.1 ± 2
Above 20	4.8 ± 1.6	4.1 ± 1.1	4.8 ± 1.9	4.7 ± 1.6	3.6 ± 0.9	4.8 ± 2.1
*P* value	**0.001**	**< 0.001**	**0.002**	0.43	0.49	**0.004**
Health condition						
Healthy	4.0 ± 1.9	3.5 ± 1.3	4.1 ± 2.4	4.2 ± 1.7	3.3 ± 1.2	4.1 ± 2.1
Diabetes	5.3 ± 1.5	4.3 ± 0.6	4.8 ± 2.1	5.3 ± 1.6	3.9 ± 0.9	4.8 ± 1.8
Heart disease	4.1 ± 1.7	3 ± 0.6	5.5 ± 1.8	5.3 ± 0.5	2 ± 0	5.6 ± 2.8
Hypertension	4.1 ± 1.9	3.4 ± 1.2	4 ± 2.2	4.7 ± 1.5	3.7 ± 0.9	4.5 ± 2.3
High cholesterol	4.1 ± 1.1	3.6 ± 1.2	4.2 ± 2.6	5.1 ± 1.5	3.8 ± 0.6	4.8 ± 1.9
*P* value	**0.03**	**0.02**	0.41	**0.006**	**< 0.001**	0.22
Smoking cigarette						
Smoker	4.4 ± 1.9	3.6 ± 1.3	4.6 ± 2.4	4.4 ± 1.5	3.2 ± 0.8	3 ± 1.4
Past smoker	4.6 ± 1.7	3.8 ± 1.3	4.6 ± 2.4			
Non-smoker	4.1 ± 1.8	3.5 ± 1.1	3.9 ± 2.2	4.6 ± 1.7	3.5 ± 1.1	4.4 ± 2.1
*P* value	0.24	0.63	0.12	0.79	0.51	0.13
Smoking Shisha						
Regular smoker	4.6 ± 1.4	4.1 ± 1.1	4.1 ± 2.3	4.7 ± 1.5	3.5 ± 1.2	4.2 ± 2.2
Never	4.3 ± 1.8	3.7 ± 1.1	4.5 ± 2.2	4.5 ± 1.7	3.5 ± 1.1	4.3 ± 2
Rarely smoker	3.7 ± 2.1	3.1 ± 1.4	3.3 ± 2.5	4.6 ± 1.4	3.5 ± 1.1	4.4 ± 2.4
*P* value	0.14	**0.01**	**0.01**	0.98	0.97	0.98
Moderate physical activity						
Never	4.1 ± 2.1	3.9 ± 1.3	5 ± 2.7	4.8 ± 1.3	3.6 ± 1.1	3.7 ± 1.7
Rarely	4.1 ± 1.9	3.6 ± 1.2	3.6 ± 2.3	4.5 ± 1.7	3.3 ± 1	4.6 ± 2.1
1–2 per week	4.2 ± 1.8	3.5 ± 1.2	4.3 ± 2.3	4.3 ± 1.9	3.5 ± 1.1	4.1 ± 2
3–4 per week	4.6 ± 1.7	3.7 ± 1.1	4.2 ± 2.2	4.7 ± 1.5	3.8 ± 1.1	4.6 ± 2.1
More than 5 per week	4 ± 1.8	2.9 ± 1.9	5.1 ± 2.2	4.8 ± 2.3	3.1 ± 1.9	4.5 ± 2.8
*P* value	0.49	0.27	0.19	0.76	0.32	0.38
Sever activity						
Never	4.4 ± 1.6	3.8 ± 1	4.1 ± 2.4	4.3 ± 1.8	3.4 ± 1	4.3 ± 2.1
Rarely	4.1 ± 1.8	3.7 ± 1.1	4.3 ± 2.2	4.7 ± 1.6	3.5 ± 1.1	4.5 ± 2.1
1–2 per week	4.4 ± 1.8	3.6 ± 1.1	5.1 ± 2.1	4.6 ± 1.7	3.6 ± 1.2	4.3 ± 2.2
3–4 per week	4 ± 2.1	3 ± 1.7	3.3 ± 2.7	5.1 ± 1.1	3.6 ± 1.2	3.8 ± 1.7
More than 5 per week	3.4 ± 2.5	2.6 ± 1.6	3.2 ± 1.9	5	2	4
*P* value	0.72	**0.03**	0.07	0.57	0.54	0.93

T1DM: type 1 diabetes mellitus

A linear regression analysis was performed

Female teachers holding bachelor’s degrees had the highest attitude score (3.6 ± 1.1) than those holding diplomas or postgraduate degrees (3.1 ± 1.2 and 3 ± 1.4, respectively; *P* = 0.02). Years of teaching experience were found to be positively associated with knowledge, attitude, and practice scores (*P* ≤ 0.002). Male teachers with more than 20 years of experience had higher knowledge, attitudes, and practice scores than the other groups. Among female teachers, only the practice score showed a significant association with teaching experience years, as female teachers with more than 20 years of teaching experience had the highest practice score (*P* = 0.004).

In addition, diabetic teachers had the highest knowledge (*P* = 0.03) and attitude (*P* = 0.02) scores compared to non-diabetic teachers. Among female teachers, diabetic teachers and those with heart diseases had the highest attitude scores (*P* = 0.006), while only diabetic teachers had the highest practice scores (*P* < 0.001). Smoking shisha status was associated with attitude and practice scores only among male teachers. Teachers who never smoked had the highest practice scores (4.5 ± 2.2) as compared to those who smoked regularly (4.1 ± 2.3) or rarely (3.3 ± 2.5) (*P* = 0.01). However, teachers who smoked regularly (4.1 ± 1.1) had the highest attitude scores (3.7 ± 1.1); while teachers who smoked rarely smoker has attitude scores of 3.1 ± 1.4 (*P* = 0.01). Physical activity levels were also found to be associated with attitude scores among male teachers with inactive teachers (3.7 ± 1.1) scoring higher than active teachers (*P* = 0.03).

## Discussion

This study attempted to examine the knowledge, attitudes, and practices with respect to T1DM among Saudi public school teachers living in Jeddah, stratify the data based on gender, and identify the associated factors. The study revealed that male school teachers were married, holding postgraduate certificates, cigarette and shisha smokers, and physically active compared to female school teachers. Overall, this study revealed moderate knowledge, favorable attitudes, and poor practices related to T1DM among school teachers in Jeddah. Female teachers were knowledgeable than male teachers about the prevalence of T1D in children and symptoms that impact diabetic students. However, male teachers had better attitudes toward having children with diabetes in their classes and attending training programs to educate themselves about handling the disease. In addition, the results indicated that knowledge, attitudes, and practice scores related to T1DM were significantly associated with advanced age, extended years of teaching experience, and diabetes in both genders. Male teachers who were married, shisha smokers, and not active had better attitudes and practices toward T1DM. Similarly, female teachers who held bachelor’s certificates had better attitudes toward T1DM.

Adequate knowledge, favourable attitudes, and good practices related to T1DM among school teachers are crucial for supervision of diabetic students who spend a long time in schools on a daily basis [12]. Inadequate care of diabetic students can result in life threatening complications such as hypoglycemia [13]. Hence, awareness in school teachers are vital in the school environment to support unwell students. In the current study, a moderate level of knowledge related to T1DM was found in some aspects, as almost one-third of school teachers knew that T1DM can affect students and half of them knew that T1DM is treated with insulin. However, higher level of knowledge was demonstrated in other areas, including T1DM complications such as polyuria, fatigue, hypoglycemia, and lack of concentration among three-quarters of school teachers. A previous study reported poor knowledge regarding T1DM complications among school teachers in Bahrain [22], whereas another study indicated the opposite [23]. The results of our study might be due to the high level of education of the school teachers included. Previous studies have shown that teachers with university certificates have higher knowledge of diabetes [5, 13, 22]. Moreover, diabetes in teachers was a significant factor associated with higher knowledge and attitude scores toward T1DM in both sexes, which can be explained by the high prevalence of the disease in the Kingdom [24]. A previous study conducted in Riyadh reported similar results [25]. In addition, previous studies have indicated that teachers who had diabetic relatives had better knowledge about the disease than other teachers [5, 22, 26]. A study examined the knowledge toward diabetes among two groups of patients; one group was diabetic and the other group was non-diabetic. The results indicated that diabetic patients had knowledge toward diabetes (32%) higher than the non-diabetic patients (25%) [25], which is consistent with our findings. In addition, two quarters of teachers said that diabetic children were eligible to attend the physical education session and almost half knew that diabetic students should take sweets or juices before physical activity class, although there were no significant differences between males and females. Previous studies have reported similar findings [5].

knowledge scores for T1DM significantly increased with age and years of experience in teaching, which is consistent with other studies conducted in Riyadh, Al-Jouf, Makkah, and Al Ahsa cities, Saudi Arabia [5, 13, 20, 25]. In general, the current study showed a moderate level of knowledge related to T1DM among school teachers in Jeddah. Similar results have been reported at the regional level, such as in Turkey [26], and on national levels including Riyadh, Mkkah, Al-Jouf, and Al Ahsa [5, 13, 20, 25]; however, evidence from Bahrain and Spain reported poor levels of knowledge of T1DM among school teachers [22, 27]. Furthermore, female teachers were more knowledgeable about T1DM than male teachers were. Previous studies have reported similar findings [20–22, 28]; however, other studies have shown the opposite [13, 25]. Actually, numerous studies have revealed gender differences in health-related knowledge and adopting healthy behaviours, as females have been proposed to be more enthusiastic in seeking health information from different sources than males [29, 30], which explains our findings.

The present study indicated favorable attitudes toward T1DM among school teachers in general and male teachers in particular. Favorable attitudes toward T1DM are in consistency with those of numerous other studies in Jordan [28], Abha [21], Al-Jouf [5], and Riyadh [25]; however, some studies showed unfavourable attitudes toward T1DM among teachers [31]. Male teachers had significantly more positive attitudes toward having children with diabetes in their classes than did female teachers. These differences might be explained by female teachers’ concerns and fear of taking responsibility for students’ health and safety more than male teachers. Thus, the unwillingness to have diabetic children in their classes might be related to anxiety and low self-confidence in their ability to effectively manage diabetic students. Furthermore, previous studies showed that almost 91% of teachers were willing to accept diabetic students [25, 26]. Another study [21] showed that 78% of teachers were willing to accept diabetic students in their classes which is essentially higher than the results reported in this study. Additionally, previous evidence showed a lack of confidence in school teachers caring for students with diabetes [32].

Furthermore, male teachers had significantly favorable attitudes toward attending training programs to educate themselves about handling diabetic students, compared to their female counterparts. Almost 73% of males said yes to attending training programs versus only 52% of females. A study in Al-Jouf stated that 93% of teachers agreed to attend training programs to enhance their awareness of diabetes which is a higher percentage than reported in this study [5]. Certainly, the findings of the present study indicate a negative relationship between knowledge and attitudes toward T1DM among teachers of both genders, as female teachers who were knowledgeable about T1D had unfavorable attitudes toward T1DM and vice versa. In addition, in the present study, more than 90% of school teachers from both genders agreed to the importance of the presence of school nurses in supporting diabetic students. Previous studies have reported similar results [5, 21]. Moreover, being married was significantly associated with higher knowledge, attitudes, and practice scores toward T1DM among male teachers only. This might be because married teachers are responsible for family; hence, they adopt healthy behaviours and favourable attitudes and seek more accurate health-related information than single teachers for the benefit of their families. In addition, the current study revealed that regular shisha smoking and no physical activity were significantly associated with positive attitudes toward T1DM among male teachers. Previous research showed that in diabetic adults who had positive attitudes toward healthy diet and physical activity, these preferences did not transcribe to their attitude towards diabetic children [33]; thus, indicating that positive attitudes, by itself, cannot impact health behaviours, which might explain our results.

Regarding T1DM management practices, the current study showed poor overall practice scores for T1DM in Jeddah. In fact, the practices of school teachers in general were fair; however, school management practices regarding T1DM were poor. For example, more than half of the teachers agreed that T1DM affects students’ academic performance and increases the absence rate, and three-quarters of them admitted to giving support to diabetic children in their classes. On the other hand, less than 10% of teachers reported special meals for diabetic students in school, and less than half of teachers reported the presence of professional support for diabetic students in school. Similarly, only a quarter of teachers said that there were trained individuals to check blood sugar and manage diabetic emergencies in their school. Hence, the current findings reflect the importance of improving schools’ attempts to better manage diabetic students. A study in Spain revealed that 50% of teachers believe that schools are unwilling to handle T1DM emergencies among affected students [34]. In addition, schools should provide appropriate training programs for teachers to educate them about T1DM. In the present study, almost 22% of men and 18% of women stated that schools provided training programs for diabetic students. A previous Saudi study reported similar findings [5], while another Saudi study reported a relatively lower percentage of training programs for teachers (28%) [21]. In addition, poor practice scores related to T1DM among Saudi teachers have been reported in a previous study [31].

Indeed, the main focus of this study was to examine the knowledge, attitudes, and practice with respect to T1DM among Saudi male and female public school teachers who are an integral part of the education system and play an important role in allowing diabetic students to develop optimally in all aspects including physically, emotionally, and academic achievement. Thus, achieving this goal will help us to understand the sources of any variances between males and females and provide accurate recommendations to improve their diabetes managements. Based on the current study results, effective educational programmes should be dedicated to school teachers especially males to enhance their knowledge about the signs and symptoms of diabetes and how to manage diabetes and its complications to take care of diabetic students in schools. Also, further research is needed to explore the reasons behind female teachers unwillingness to have diabetic children in their classes. Furthermore, encourage female teachers to participate in training programs to educate themselves about handling diabetic students and to increase their confident in applying their knowledge in how to manage diabetes and its complications to take care of diabetic students. Moreover, improving schools’ efforts to better manage diabetic students is crucial including the presence of trained individuals to check blood sugar and manage diabetic emergencies in schools and provide appropriate training programs for teachers to educate them about diabetes management.

This study had some strengths and limitations that should be addressed. First, recall bias might be an issue, as school teachers were asked to remember some information. The cross-sectional study design is another limitation because it shows an association, but not causality. In addition, data were collected through an online survey which might have introduced selection bias. In contrast, pretested and validated surveys were used in this study to collect the required data which is one of the strengths of the study. Furthermore, the findings are believed to be representative of school teachers in Jeddah, as teachers included in this study were from the six educational offices in the city; however, the findings cannot be generalized to all teachers in Saudi Arabia. Moreover, few studies have examined the knowledge, attitudes, and practice with respect to T1DM among school teachers in Saudi Arabia [5, 13, 20, 25]. Nevertheless, none of these studies were conducted in Jeddah city and assessed gender differences regarding knowledge, attitudes, and practice toward T1DM among school teachers which is another strength of this study.

## Conclusions

This study showed moderate knowledge, favorable attitudes, and poor practices related to T1DM among school teachers in Jeddah and significant gender differences with regard to knowledge, attitudes, and practices. Furthermore, the results indicated that knowledge, attitudes, and practice scores toward T1DM were significantly associated with advanced age, extended years of teaching experience, and being diabetic in both sexes. Therefore, this study recommends that more efforts should be made to improve the knowledge, attitude, and practice of school teachers by conducting an effective nutritional education initiative regarding diabetes care with official authorities such as the Ministry of Health and the Food and Drug Authority within the school. Furthermore, schools should provide teachers with standardized training programs to recognize and respond to the signs and symptoms of diabetes and how to manage diabetes and its complications to take care of diabetic students. Teachers should also be provided with guidance during emergencies to support students with diabetes as needed, especially female teachers, to increase their self-confidence in their ability to effectively manage diabetic students. In addition, it is essential for the safety of diabetic students in schools to have diabetes equipment and supplies as first aid to manage students’ diabetes. Moreover, it is important to have constructive and regular communication between home and school to help enhance a good relationship between parents and school staff to effectively manage diabetic students. Finally, further research is needed to explore the factors that could motivate teachers and build their self-efficacy in managing diabetes in schools.

## Supplementary Information

**Additional file 1.**

## Data Availability

The datasets generated and/or analysed during this study are not publicly available because of the use of data for further publications but are available from the corresponding author upon reasonable request.

## Abbreviations

T1DM: type 1 diabetes mellitus
