# Supplementary material for: Gender differences in knowledge, attitudes, and practices with respect to type 1 diabetes among Saudi public-school teachers
Source: BMC Public Health. 2023 Jan 17;23:118. doi: 10.1186/s12889-023-15043-w (PMC9847176; doi:10.1186/s12889-023-15043-w)
Supplement: Supplementary file 1 — Additional file 1. [file 12889_2023_15043_MOESM1_ESM.docx]

**Additional file 1**

Knowledge of T1DM:

1. Students can have type 1 diabetes?

□Yes □No □Not sure

1. T1DM leads to polyuria in diabetic student?

□Yes □No □Not sure

1. T1DM leads to fatigue and lack of concentration in diabetic student?

□Yes □No □Not sure

1. T1DM leads to loss of weight in diabetic student?

□Yes □No □Not sure

1. T1DM is treated with insulin?

□Yes □No □Not sure

1. Tremors and sweating means hypoglycaemia in diabetic student?

□Yes □No □Not sure

1. The diabetic student should take sweets or juices before physical activities class?

□Yes □No □Not sure

Attitudes toward T1DM:

1. Are you willing to have diabetic children in your class?

□Yes □No □Not sure

1. In case of hypoglycaemia, should the diabetic student take sweet juice?

□Yes □No □Not sure

1. In case of coma, can small amount of jam or honey be put into the mouth of the diabetic student?

□Yes □No □Not sure

1. Would you like to join training program for dealing with diabetic students?

□Yes □No □Not sure

1. Do you support presence of school nurse?

□Yes □No □Not sure

Practices related to T1DM:

1. T1DM affects the student’ academic performance?

□Yes □No □Not sure

1. T1DM increases absence rate of diabetic student?

□Yes □No □Not sure

1. Do you have any diabetic student in your class?

□Yes □No □Not sure

1. Do you give support to the diabetic children in your class?

□Yes □No □Not sure

1. Are diabetic children eligible to attend the physical education session?

□Yes □No □Not sure

1. Does your school present special meals for diabetic student?

□Yes □No □Not sure

1. Does your school appoint somebody to look after the diabetic students?

□Yes □No □Not sure

1. Is there any trained person to check blood sugar and inject insulin in your school?

□Yes □No □Not sure

1. Is there any trained person in dealing with diabetic emergencies in your school?

□Yes □No □Not sure

1. Does your school have a training program for dealing with diabetic students?

□Yes □No □Not sure
